# Regression models for monitoring trace metal accumulations by *Faba sativa* Bernh. plants grown in soils amended with different rates of sewage sludge

**DOI:** 10.1038/s41598-019-41807-9

**Published:** 2019-04-01

**Authors:** Ebrahem M. Eid, Sulaiman A. Alrumman, Tarek M. Galal, Ahmed F. El-Bebany

**Affiliations:** 1 Biology Department, College of Science, King Khalid University, Abha, 61321 P.O. Box 9004, Saudi Arabia; 2 Present Address: Botany Department, Faculty of Science, Kafr El-Sheikh University, Kafr El-Sheikh, 33516 Egypt; 3 Botany and Microbiology Department, Faculty of Science, Helwan University, Cairo, Egypt; 4 Plant Pathology Department, Faculty of Agriculture, Alexandria University, El-Shatby, 21545 Alexandria, Egypt

## Abstract

The present study was conducted using a pot experiment to develop regression models for the prediction of trace metal concentrations in faba bean (*Faba sativa* Bernh.) plants cultivated in soils amended with different rates of sewage sludge to monitor possible human health risks. The trace metal concentrations in the different tissues of faba bean showed that most of the investigated trace metals were accumulated in the plant roots rather than in the other tissues. Meanwhile, the fruits accumulated the lowest concentration of most trace metals. The trace metal concentrations of the faba bean plants had a significant positive correlation with the organic matter content and a significant negative correlation with the soil pH. Transfer of trace metals from the soil to faba bean roots indicated that Al, Cu, Pb and Zn had a transfer factor that exceeded one, whereas the TF of the investigated trace metals from the roots to the fruits did not exceed one. The daily intake rate of the investigated trace metals did not exceed one in both adults and children. On the other side, the hazard quotient of trace metals from consuming faba bean fruits had values <1 for most investigated trace metals except Al and Mn in adults and in children. It is worth mentioning that the predicted trace metal concentrations via the established regression models and measured values from the validation data set were not significantly different (*P* > 0.05). Therefore, these developed models will be useful for prediction of trace metals uptake by faba bean grown in soil amended with sewage sludge so possible human risks can be identified.

## Introduction

Sewage sludge is an organic byproduct of the treatment of municipal or industrial wastewater that has significant amounts of macro- and micro-nutrients^1^. The most economical and helpful choices for sewage sludge elimination are land application and landfilling^2^. Amending agricultural soils with this valuable organic fertilizer improves the physico-chemical properties of the soil, including fertility and therefore, crop yield^3–7^. On the other hand, sewage sludge could have high trace metal contents as a result of inputs from human activity, such as the use of fertilizers, human excreta, domestic water from baths, showers and dishwashing, mine drainage, runoff water from roofs and roads, and industrial wastewaters^5,8–10^. Consequently, the long-term application of sewage sludge as an amendment material could result in trace metal accumulation in the soil^11^. Moreover, the effect of sewage sludge on soil reactions is translated directly into the bio-availability of soil trace metals^12^, where plants grown in soils amended with sewage sludge accumulated higher concentrations of trace metals compared to those grown in un-amended soil^13^.

Trace metals are identified as cumulative, intractable in-organic pollutants widely existing in the environment, and their effects of pollution manifest as trace metal migration and transformation^14^. These pollutants are elements that cannot be degraded by microbial or chemical process to alter or reduce their toxicity over time^15^. The toxicity of trace metals depends on their concentrations and availability and on the target organism; however, the serious adverse health effects would be minimized if these trace metals were reduced to transfer to the food chain^16^. Recently, there has been an increasing concern over the accumulation of trace metals in food and their potential hazards to human health^14,17,18^. The transfer and accumulation of trace metals in the animal and human bodies over the food chain cause DNA damage, carcinogenic effects and induction of mutations^19,20^. Other than safety concerns, excessive trace metals also contaminate soils and affect crop growth and quality^17^. Therefore, it is essential to understand the transfer of trace metals between soil and plants, and some models have been created to characterize the transfer of these trace metals from soil to plants^18^. In addition, it is of importance to develop a relationship between the trace metal concentrations in soils and their transmission to edible plant parts based on the field data to improve the standards of soil environmental quality^17^.

Crop plants contribute greatly to human trace metal exposure; therefore, crop-specific prediction models (such as regression models for cucumber^21^, rice^22^, spinach^16^, tomato^23^ and wheat^24^) are a reasonable tool for estimating the potential dietary dangers of trace metals^25^. Empirical models are frequently used in practical applications such as soil remediation and risk assessment^26^. These models can be of great value in the evaluation of potential risks to the environment posed by different trace metals, and they are frequently used to predict the uptake and accumulation of these trace metals by crop plants^27^. A simple estimation of the transfer factors can help as a rough insight into the range of trace metal uptake, but it does not consider site-specific conditions in detail; thus, the regression models, in which the concentrations of trace metals in plants are predicted by various soil parameters, mostly the total concentration of a trace metal in the soil, soil pH, and organic matter content^28^, are useful tools for estimating the trace metal concentrations in crop plants. Recently, regression analysis has been vastly used in numerous environment-related areas as a suitable way for experimental data analysis^16,21,29^.

*Faba sativa* Bernh. (faba bean), *Fabaceae*, is an important pulse crop, and it is considered one of the major food legume crops in the Mediterranean region, the Middle East and Latin America^30^. It is adapted to grow in a broad zone of climates, and it is a perfect rotation crop because its roots are associated with nitrogen-fixing bacteria^31^. Globally, it is the third most significant feed legume after soybean and pea^32^. Faba bean is a valuable protein-rich food that provides a large sector of human populations in developing countries with a cheap source of protein, vitamins, starch, minerals and anti-oxidant components^33^. In the our previous study^6^, the suitability of the amendment with sewage sludge for faba bean was evaluated by assessing the soil properties, plant growth measurements and trace metal accumulation in faba bean grown at different sewage sludge amendment rates under a greenhouse condition. To our knowledge, so far, no prediction models for the uptake of trace metals by faba bean plants grown on soil amended with sewage sludge can be found in the literature. Thus, the present study was conducted to develop a novel regression models for predicting the concentrations of trace metals in faba bean plants from their concentration in the soil by using the organic matter content and soil pH as co-factors. These models will be useful for prediction of trace metals uptake by faba bean so possible human risks can be identified.

## The Experiment

### Plant cultivation

Sewage sludge was sampled from the Abha wastewater treatment plant, which produces approximately 90 ton day^−1^ from treating approximately 41.3 × 10^3^ m^3^ wastewater day^−1^ (ref. ^4^); meanwhile, soils were collected from a 0–20 cm profile from the adjacent agricultural lands. The sewage sludge used in the current study was acidic and had a high content of organic matter compared with non-amended soil which collected from agricultural lands (Supplementary Material 1). The soil and sewage sludge samples were air dried, ground using an Agate mortar and sieved through a 2 mm sieve. After that, sewage sludge was mixed with soil at rates of 0 (control), 10, 20, 30, 40 and 50 g kg^−1^. A completely random design including 36 plastic pots (6 L volume), using 6 replicates per treatment, was constructed. Each pot was filled with 4 kg soil, and then 5 faba bean seeds were sown in each pot and left to grow for 80 days in the greenhouse under natural day/light conditions. Periodic irrigation was applied to maintain a uniform moisture level in each pot, and the plants were thinned to 2 plants per pot after fifteen days from sowing; meanwhile, the emerged weeds were weeded manually.

### Elemental determination

After harvesting faba bean plants, the amended soil samples were air dried and passed through a 2-mm sieve for estimating the organic matter content by loss-on-ignition at 550 °C for 2 hours^34^. The soil reaction (pH) was determined in a prepared soil extract (1:5) using the pH meter Model 9107 BN, ORION type^35^. The harvested plants were divided into roots, stems, leaves and pods and dried at 60 °C until a constant weight was attained. Then, the plant materials were ground using a metal-free plastic mill and saved for the subsequent analyses. A microwave sample preparation system (PerkinElmer Titan MPS, PerkinElmer Inc., USA) was used to extract 1 g ground soil and plant sample by adding 20 ml tri-acid mixture of HNO_3_:H_2_SO_4_:HClO_4_ (5:1:1, *v/v/v*) until a transparent colour appeared. Then, the digests were filtered and diluted to 25 ml with double de-ionized water^35^. Analytical-grade chemicals were used for sample digestion, and blank samples were involved to confirm the accuracy and precision of the analyses. An atomic absorption spectrophotometer (Shimadzu AA-6300; Shimadzu Co. Ltd., Japan) was used to determine the concentrations of Al, Co, Cr, Cu, Fe, Mn, Ni, Pb and Zn. All of these procedures are outlined by Allen^35^. The detection limits of trace metals (in µg l^−1^) were as follows: 5.0 for Fe; 1.5 for Cu, Mn and Zn; 20.0 for Al; 15.0 for Pb; 9.0 for Co; 3.0 for Cr and 6.0 for Ni. All detection limits were established on a 98% confidence level (3 standard deviations). The instrument setting and operational conditions followed the manufacturers’ specifications.

### Data analysis

The data of pH, organic matter content, trace metals of the soil amended with sewage sludge and trace metal concentrations in the tissues of faba bean plants were presented as minimum, maximum, mean and coefficient of variance (CV; the ratio of the standard deviation to the mean). The transfer factor (TF) is an appropriate method to model the transfer of trace metals from soil to plants. It is based on the hypothesis of a linear relationship between plant and soil trace metal concentrations. The TF is used to assess the potential of a plant to accumulate trace metals in its below-ground tissues and translocate them to the above-ground tissues. It was calculated following Galal *et al*.^36^:

$${{\rm{TF}}}_{roots}={{\rm{C}}}_{roots}{/C}_{soil}$$

$${{\rm{TF}}}_{stems}={{\rm{C}}}_{stems}/{{\rm{C}}}_{roots}$$

$${{\rm{TF}}}_{leaves}={{\rm{C}}}_{leaves}/{{\rm{C}}}_{roots}$$

$${{\rm{TF}}}_{fruits}={{\rm{C}}}_{fruits}/{{\rm{C}}}_{roots}$$

where C_*soil*_, C_*roots*_, C_*stems*_, C_*leaves*_ and C_*fruits*_ are the trace metal concentration (mg kg^−1^) in the soil, roots, stems, leaves and fruits, respectively. The daily intake rate (DIR) of trace metals through the consumption of faba bean fruits was calculated according to the given equation^37^:

$${\rm{DIR}}={{\rm{C}}}_{tracemetal}\times {{\rm{C}}}_{factor}\times {{\rm{D}}}_{{int}ake}/{\rm{BW}}$$

where C_*trace metal*_ is the average trace metal concentration in the edible parts (fruits) of faba bean (mg kg^−1^); C_*factor*_ is a factor of 0.085 was used to convert the fresh to dry weight of these green fruits^38^; D_*intake*_ is the daily intake of faba bean (0.345 and 0.232 kg person^−1^ day^−1^ FW) for adults and children, respectively; and BW is the average body weight (60.0 and 32.7 kg) for adults and children, respectively. The hazard quotient (HQ) for the consumers through the consumption of contaminated faba bean was assessed as^39^:

$${\rm{HQ}}={\rm{DIR}}/{{\rm{R}}}_{f}{\rm{D}}$$

where R_*f*_D is the reference dose of trace metals. The values of R_*f*_D for Al, Co, Cr, Cu, Fe, Mn, Ni, Pb and Zn were used as 0.070^40^, 0.043^39^, 1.500^39^, 0.040^41^, 0.700^41^, 0.014^41^, 0.020^40^, 0.001^39^ and 0.300^41^ mg kg^−1^ BW day^−1^, respectively. Moreover, the assessment of the health risk from trace metals was calculated based on Hazard Index (HI) values as follows^42^:

$${\rm{HI}}=\sum {{\rm{HQ}}}_{i}{(}_{i}{\rm{is}}\,{\rm{the}}\,{\rm{HQ}}\,{\rm{of}}\,{\rm{each}}\,{\rm{trace}}\,{\rm{metal}})$$

The values of HQ and HI < 1 are considered as no risk, but if the values are >1, then they are considered to high risk of the trace metals with long term health hazard effects^43^.

The relationship between faba bean trace metals and soil variables was assessed by calculating the simple linear correlation coefficient (*r*). Eighteen observations from the dataset for each of roots, stems, leaves and fruits were selected for use as validation datasets; meanwhile, eighteen observations each for roots, stems, leaves and fruits were used in the regression procedure, where regression models were created to predict the trace metal concentrations in faba bean tissues from their concentration in the soil and using the organic matter content and soil pH as co-factors. The model quality was determined on the basis of the coefficient of determination (*R*^2^), the model efficiency (ME) and the model strength (determined by the mean normalized average error, MNAE), which were calculated as follows:

$${\rm{M}}{\rm{E}}=1-\{\phantom{\frac{2}{1}}\,\,\sum {({{\rm{C}}}_{model}{\textstyle \text{-}}{{\rm{C}}}_{measured})}^{2}\,/\sum ({{\rm{C}}}_{measured}{\textstyle \text{-}}{{\rm{C}}}_{mean}{)}^{2}\,\phantom{\frac{2}{1}}\}$$

$${\rm{MNAE}}=\{\,\sum (|{{\rm{C}}}_{{model}}\mbox{--}{{\rm{C}}}_{measured}|{/C}_{measured})\}/n$$

where C_*model*_ is the predicted trace metal concentration given by the model, C_*measured*_ is the measured trace metal concentration, C_*mean*_ is the mean measured trace metal concentration, and *n* is the number of observations. The estimated concentration of a trace metal in a tissue was compared with the measured trace metal in the same tissue using Student’s *t*-test. SPSS 15.0 was used to process all of the statistical analyses^44^.

## Results

### Characteristics of soil-sewage sludge

The soil analysis data indicated that the soil-sewage sludge combination supporting faba bean cultivation was alkaline, where the pH ranged from 8.39 to 9.04 with a mean of 8.69 (Table 1). In addition, this soil was characterized by relatively low organic matter content with a mean value of 1.8%. The trace metal concentrations of the sewage amended soil fell in the following order: Fe > Al > Mn > Cr > Zn > Ni > Co > Cu > Pb. The mean concentration of Al, Cu and Pb was characterized by high coefficients of variance (25.1, 25.7 and 35.3%).

**Table 1:** Minimum, maximum, mean and coefficient of variance (CV) of the pH, organic matter content and trace metals of the soil amended with sewage sludge after harvesting faba bean plants grown for 80 days.

Value	pH	Organic matter content (%)	Trace metal (mg kg^−1^)								
			Al	Co	Cr	Cu	Fe	Mn	Ni	Pb	Zn
Minimum	8.39	0.8	5506.1	34.5	196.1	13.4	20619.8	660.0	50.8	6.3	67.6
Maximum	9.04	2.8	11596.4	57.9	289.9	33.6	27789.1	808.8	78.1	22.4	90.9
Mean (*n* = 36)	8.69	1.8	7263.5	46.7	270.4	19.0	25758.4	757.5	62.4	11.8	77.4
CV (%)	2.3	26.7	25.1	11.9	8.5	25.7	6.0	4.9	10.2	35.3	8.2

### Trace metal contents in plant tissues

The trace metal concentrations in the different tissues of faba bean showed that most of the investigated trace metals except Al, Co and Pb were accumulated in the plant roots rather than in the other tissues (Table 2). The highest mean concentration of trace metals in the roots was recorded for Fe, followed by Mn, Cr, Zn, Ni and Cu (15492.1, 555.6, 133.6, 102.8, 57.6 and 27.7 mg kg^−1^, respectively). Meanwhile, the highest concentrations of Al, Co and Pb (17811.3, 46.6 and 35.1 mg kg^−1^) were found in the plant leaves. The fruits of faba bean accumulated the lowest concentration of most trace metals except Cu, Mn and Zn (8.5, 47.8 and 25.3 mg kg^−1^), which were found in the plant stems. Notably, the trace metal concentrations in the edible fruits fell in the following order: Fe > Al > Mn > Zn > Cu > Co > Ni > Cr > Pb.

**Table 2:** Trace metal concentrations in the tissues of faba bean plants grown for 80 days in soil amended with sewage sludge. CV: coefficient of variance.

Tissue	Value	Trace metal concentration (mg kg^−1^)								
		Al	Co	Cr	Cu	Fe	Mn	Ni	Pb	Zn
Roots	Minimum	11109.3	11.5	81.8	15.4	10474.3	297.0	36.6	12.9	37.0
	Maximum	19333.8	27.5	192.6	44.1	19556.4	788.4	70.8	37.3	166.9
	Mean (*n* = 36)	15962.0	19.8	133.6	27.7	15492.1	555.6	57.6	27.8	102.8
	CV (%)	15.3	20.5	22.9	33.1	15.7	27.2	18.4	28.4	39.1
Stems	Minimum	13348.1	32.6	4.5	5.6	425.5	33.4	45.3	26.7	12.3
	Maximum	19086.6	46.0	7.7	11.3	744.6	68.4	62.4	38.3	35.5
	Mean (*n* = 36)	16919.1	40.2	5.9	8.5	617.0	47.8	55.9	33.9	25.3
	CV (%)	10.1	10.0	12.3	21.5	11.6	24.5	9.4	10.3	28.2
Leaves	Minimum	15755.1	43.2	4.5	7.4	344.0	107.0	45.8	30.7	23.8
	Maximum	19226.1	51.2	7.8	14.6	1165.8	367.7	60.8	38.3	58.9
	Mean (*n* = 36)	17811.3	46.6	6.2	10.6	812.7	248.9	52.8	35.1	40.6
	CV (%)	5.4	4.8	16.7	21.4	29.9	35.0	9.1	6.1	23.3
Fruits	Minimum	139.3	8.3	1.2	12.7	202.6	40.9	4.7	0.4	34.7
	Maximum	492.2	14.5	4.4	23.6	1212.6	123.6	10.9	2.5	69.0
	Mean (*n* = 36)	231.4	10.9	2.0	17.2	377.1	77.3	6.8	0.9	48.5
	CV (%)	43.5	13.0	45.9	16.3	65.3	35.3	28.8	66.7	19.8

### Correlation coefficient

The simple linear correlation coefficient between soil variables and trace metal concentrations in the different tissues of faba bean indicated that the increase in soil organic matter content significantly increased the trace metal concentrations of the different plant tissues (Table 3). On the contrary, the soil pH had a significant negative effect on the plant trace metal concentrations. Moreover, it was found that most of the correlation coefficients between soil trace metal concentrations and the corresponding plant trace metals were significantly positive except for some coefficients such as soil Co with fruit Co; soil Cr with fruit Cr; and soil Fe with stem Fe.

**Table 3:** Pearson correlation coefficient (*r*-value, *n* = 36) between trace metals in faba bean tissues and their concentrations in soil. OM: organic matter.

Plant trace metals	Soil trace metals									Soil pH	Soil OM content
	Al	Co	Cr	Cu	Fe	Mn	Ni	Pb	Zn		
***Roots***											
Al	0.43**	0.31	0.64***	0.42*	0.51**	0.66***	0.50**	0.40*	0.63***	−0.74***	0.53**
Co	0.72***	0.58***	0.40*	0.61***	0.33	0.28	0.76***	0.59***	0.59***	−0.60***	0.49**
Cr	0.80***	0.58***	0.65***	0.71***	0.52**	0.56***	0.81***	0.75***	0.61***	−0.83***	0.67***
Cu	0.80***	0.55**	0.60***	0.70***	0.50**	0.58***	0.80***	0.69***	0.68***	−0.86***	0.69***
Fe	0.70***	0.61***	0.73***	0.57***	0.56***	0.58***	0.77***	0.73***	0.52**	−0.75***	0.75***
Mn	0.75***	0.51**	0.69***	0.66***	0.58***	0.68***	0.75***	0.65***	0.66***	−0.85***	0.72***
Ni	0.55***	0.55***	0.59***	0.50**	0.47**	0.65***	0.69***	0.45**	0.53**	−0.79***	0.54**
Pb	0.49**	0.54**	0.68***	0.55**	0.68***	0.69***	0.68***	0.51**	0.61***	−0.83***	0.59***
Zn	0.73***	0.56***	0.67***	0.67***	0.59***	0.65***	0.78***	0.64***	0.67***	−0.83***	0.71***
***Stems***											
Al	0.50**	0.32	0.40*	0.56***	0.35*	0.42*	0.53**	0.47**	0.64***	−0.73***	0.50**
Co	0.57***	0.35*	0.55**	0.52**	0.39*	0.55**	0.57***	0.52**	0.61***	−0.78***	0.57***
Cr	0.79***	0.44**	0.36*	0.39*	0.27	0.19	0.61***	0.49**	0.31	−0.63***	0.43**
Cu	0.68***	0.50**	0.68***	0.68***	0.53**	0.67***	0.73***	0.67***	0.65***	−0.87***	0.66***
Fe	0.45**	0.22	0.34*	0.26	0.07	0.18	0.32	0.59***	0.10	−0.49**	0.63***
Mn	0.81***	0.53**	0.61***	0.71***	0.46**	0.58***	0.78***	0.75***	0.66***	−0.88***	0.69***
Ni	0.53**	0.33	0.41*	0.58***	0.37*	0.43**	0.55**	0.49**	0.65***	−0.75***	0.51**
Pb	0.52**	0.33*	0.40*	0.58***	0.35*	0.43**	0.55**	0.48**	0.64***	−0.74***	0.50**
Zn	0.68***	0.45**	0.77***	0.57***	0.58***	0.67***	0.678***	0.66***	0.60***	−0.82***	0.72***
***Leaves***											
Al	0.47**	0.29	0.53**	0.34*	0.54**	0.46**	0.46**	0.32	0.48**	−0.59***	0.52**
Co	0.68***	0.54**	0.42*	0.53**	0.45**	0.39*	0.66***	0.66***	0.27	−0.61***	0.78***
Cr	0.61***	0.63***	0.62***	0.57***	0.50**	0.45**	0.75***	0.62***	0.50**	−0.73***	0.63***
Cu	0.73***	0.49**	0.56***	0.67***	0.51*	0.58***	0.72***	0.58***	0.57***	−0.81***	0.67***
Fe	0.58***	0.60***	0.57***	0.60***	0.47**	0.54**	0.71***	0.48**	0.43**	−0.74***	0.59***
Mn	0.64***	0.55**	0.69***	0.62***	0.57***	0.67***	0.73***	0.60***	0.59***	−0.89***	0.68***
Ni	0.75***	0.45**	0.56***	0.69***	0.48**	0.50**	0.72***	0.63***	0.71***	−0.83***	0.51**
Pb	0.54**	0.20	0.60***	0.50**	0.53**	0.70***	0.44**	0.35*	0.50**	−0.75***	0.53**
Zn	0.80***	0.58***	0.45**	0.70***	0.38*	0.46**	0.79***	0.69***	0.55**	−0.86***	0.63***
***Fruits***											
Al	0.66***	0.48**	0.32	0.73***	0.41*	0.42*	0.68***	0.41*	0.53**	−0.70***	0.33*
Co	0.55**	0.28	0.50**	0.53**	0.29	0.62***	0.54**	0.41*	0.68***	−0.69***	0.39*
Cr	0.70***	0.55***	0.29	0.71***	0.22	0.24	0.71***	0.73***	0.45**	−0.47**	0.43*
Cu	0.54**	0.50**	0.53**	0.63***	0.58***	0.59***	0.67***	0.50**	0.61***	−0.79***	0.62***
Fe	0.76***	0.59***	0.35*	0.83***	0.39*	0.33*	0.79***	0.62***	0.57***	−0.60***	0.40*
Mn	0.66***	0.42*	0.45**	0.65***	0.45**	0.59***	0.66***	0.63***	0.65***	−0.82***	0.67***
Ni	0.81***	0.50**	0.44**	0.78***	0.36*	0.49**	0.76***	0.75***	0.64***	−0.86***	0.61***
Pb	0.93***	0.53**	0.47**	0.73***	0.35*	0.47**	0.81***	0.72***	0.61***	−0.79***	0.55**
Zn	0.55**	0.40*	0.48**	0.49**	0.36*	0.52**	0.58***	0.35*	0.55**	−0.72***	0.42*

*P < 0.05, **P < 0.01, ***P < 0.001.

### Transfer factor

**Figure 1:**
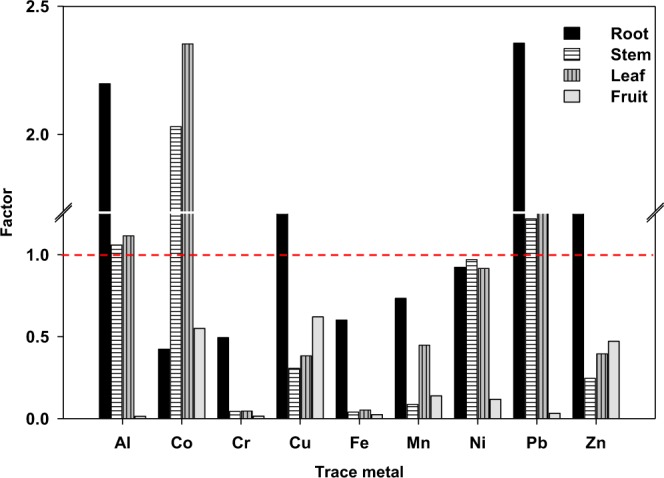
Transfer factor of trace metals from the soil to faba bean tissues.

The transfer factor (TF) assessing the uptake of trace metals by faba bean roots from the soil indicated that Al, Cu, Pb and Zn accumulated in the roots with a TF (2.20, 1.46, 2.36 and 1.33) exceeding one (Fig. 1). In addition, Al, Co and Pb were translocated from the roots to the stems and leaves with a TF of 1.06, 2.03 and 1.22 for stems; and 1.12, 2.35 and 1.26 for the leaves, respectively. Notably, the TF of the investigated trace metals from the roots to the fruits did not exceed one.

### Health risks

The daily intake rate (DIR) of the investigated trace metals did not exceed one in both adults and children (Table 4). On the other side, the hazard quotient (HQ) of trace metals from consuming faba bean fruits had values <1 for most investigated trace metals except Al and Mn (1.734 and 2.897 mg kg^−1^ day^−1^, respectively) in adults and (1.994 and 3.330 mg kg^−1^ day^−1^, respectively) in children. According to the proposed risk, the HQ can be arranged as follows: Mn > Al > Pb > Fe > Cu > Ni > Co > Zn > Cr for both adults and children. Moreover, the hazard index (HI) from consuming faba bean was found to be 6.01 and 6.91 for adults and children, respectively.

**Table 4:** Daily intake rate (DIR) and health quotient (HQ) of trace metals in faba bean plants grown for 80 days in soil amended with sewage sludge.

Trace metal	DIR (mg day^−1^)		HQ (mg kg^−1^ day^−1^)	
	Adults	Children	Adults	Children
Al	0.1131	0.1395	1.734	1.994
Co	0.0053	0.0066	0.133	0.153
Cr	0.0010	0.0012	0.001	0.001
Cu	0.0084	0.0104	0.226	0.259
Fe	0.1843	0.2274	0.283	0.325
Mn	0.0378	0.0466	2.897	3.330
Ni	0.0033	0.0041	0.178	0.205
Pb	0.0004	0.0005	0.472	0.543
Zn	0.0237	0.0292	0.085	0.097

### Regression models

Regression models were created to estimate trace metal concentrations in faba bean tissues based on their concentration in the soil by using the organic matter content and soil pH as co-factors. The results and the prediction precisions of these models are summarized in Table 5. Correlations between the measured and predicted trace metal values, with high *R*^2^ and low mean averaged errors reflected the goodness of fit of the model. As well, presence of non-significant difference (*P* > 0.05) between the measured and predicted concentrations of the trace metal means good performance of the developed model. The *R*^2^ for all models was high and ranged between 0.506 and 0.909 for fruit Cr and root Cu, respectively. The model efficiency (ME) values ranged between 0.565 and 0.911 for root Co and Mn; 0.321 and 0.864 for stem Cr and Zn; 0.298 and 0.938 for leaf Fe and Mn; and 0.405 and 0.858 for fruit Cr and Mn, respectively. In addition, the regression models had a low mean normalized average error (MNAE), which ranged between 0.046 and 0.167 for root Fe and Zn; 0.044 and 0.101 for stem Co and Zn; 0.029 and 0.167 for leaf Pb and Fe; and 0.059 and 0.459 for fruit Cu and Fe.

**Table 5:** Regression models between the trace metal concentrations in faba bean tissues and soil trace metals, pH and organic matter (OM) content.

Equation	*R* ^2^	ME	MNAE	Student’s *t*-test	
				*t*-value	*P*
***Roots***					
Al_*root*_ = 95495.319 − 0.153 × Al_*soil*_ − 9127.812 × pH + 502.614 × OM	0.742	0.679	0.092	0.497	0.640
Co_*root*_ = 60.746 + 0.279 × Co_*soil*_ − 6.479 × pH + 1.275 × OM	0.697	0.565	0.080	0.730	0.498
Cr_*root*_ = 950.114 + 0.160 × Cr_*soil*_ − 100.887 × pH + 9.341 × OM	0.851	0.870	0.082	0.589	0.581
Cu_*root*_ = 241.687 + 0.565 × Cu_*soil*_ − 26.529 × pH + 3.186 × OM	0.909	0.772	0.108	0.021	0.984
Fe_*root*_ = 56685.860 + 0.082 × Fe_*soil*_ − 5418.842 × pH + 2080.731 × OM	0.821	0.871	0.046	0.386	0.715
Mn_*root*_ = 3514.041 + 0.708 × Mn_*soil*_ − 419.334 × pH + 82.070 × OM	0.883	0.911	0.064	0.374	0.723
Ni_*root*_ = 307.267 + 0.502 × Ni_*soil*_ − 32.276 × pH − 0.254 × OM	0.825	0.727	0.111	0.679	0.527
Pb_*root*_ = 300.588− 0.120 × Pb_*soil*_ − 31.583 × pH + 1.707 × OM	0.831	0.846	0.135	1.472	0.201
Zn_*root*_ = 754.425 + 2.039 × Zn_*soil*_ − 97.864 × pH + 22.537 × OM	0.902	0.875	0.167	0.120	0.909
***Stems***					
Al_*stem*_ = 68133.330 + 0.052 × Al_*soil*_ − 5937.589 × pH + 11.590 × OM	0.728	0.607	0.068	0.143	0.892
Co_*stem*_ = 168.106 − 0.006 × Co_*soil*_ − 14.822 × pH + 0.667 × OM	0.784	0.808	0.044	0.092	0.931
Cr_*stem*_ = 27.879 − 0.004 × Cr_*soil*_ − 2.429 × pH + 0.099 × OM	0.633	0.321	0.084	1.172	0.294
Cu_*stem*_ = 57.995 − 0.095 × Cu_*soil*_ − 5.993 × pH + 0.425 × OM	0.905	0.842	0.067	0.695	0.518
Fe_*stem*_ = 1936.956 − 0.029 × Fe_*soil*_ − 92.362 × pH + 126.698 × OM	0.788	0.556	0.063	0.515	0.628
Mn_*stem*_ = 444.211 − 0.010 × Mn_*soil*_ − 45.676 × pH + 4.364 × OM	0.887	0.824	0.076	0.267	0.800
Ni_*stem*_ = 204.629 + 0.086 × Ni_*soil*_ − 17.741 × pH + 0.078 × OM	0.749	0.640	0.058	0.175	0.868
Pb_*stem*_ = 143.809 + 0.057 × Pb_*soil*_ − 12.662 × pH − 0.262 × OM	0.741	0.649	0.064	0.131	0.901
Zn_*stem*_ = 152.111 + 0.270 × Zn_*soil*_ − 17.912 × pH + 4.377 × OM	0.877	0.864	0.101	0.936	0.392
***Leaves***					
Al_*leaf*_ = 33467.206 + 0.051 × Al_*soil*_ − 1924.219 × pH + 381.985 × OM	0.620	0.344	0.044	0.354	0.737
Co_*leaf*_ = 37.147 + 0.128 × Co_*soil*_ − 0.240 × pH + 3.019 × OM	0.840	0.449	0.032	0.480	0.652
Cr_*leaf*_ = 26.198 + 0.008 × Cr_*soil*_ − 2.619 × pH + 0.389 × OM	0.766	0.301	0.086	0.292	0.782
Cu_*leaf*_ = 55.449 + 0.135 × Cu_*soil*_ − 5.665 × pH + 1.010 × OM	0.859	0.711	0.086	0.109	0.918
Fe_*leaf*_ = 7108.198 + 0.004 × Fe_*soil*_ − 752.985 × pH + 83.436 × OM	0.752	0.298	0.167	1.260	0.263
Mn_*leaf*_ = 2645.721 + 0.310 × Mn_*soil*_ − 308.062 × pH + 25.300 × OM	0.901	0.938	0.062	0.436	0.681
Ni_*leaf*_ = 185.807 + 0.242 × Ni_*soil*_ − 16.789 × pH − 1.185 × OM	0.862	0.694	0.049	1.116	0.315
Pb_*leaf*_ = 106.466 − 0.190 × Pb_*soil*_ − 8.243 × pH + 1.382 × OM	0.782	0.741	0.029	0.781	0.470
Zn_*leaf*_ = 323.331 + 0.213 × Zn_*soil*_ − 34.777 × pH + 1.692 × OM	0.873	0.847	0.068	0.673	0.531
***Fruits***					
Al_*fruit*_ = 3061.626 + 0.026 × Al_*soil*_ − 330.410 × pH −78.413 × OM	0.795	0.548	0.285	1.010	0.359
Co_*fruit*_ = 61.376 − 0.010 × Co_*soil*_ − 5.676 × pH − 0.387 × OM	0.696	0.675	0.063	0.753	0.486
Cr_*fruit*_ = 18.351 − 0.006 × Cr_*soil*_ − 1.802 × pH + 0.475 × OM	0.506	0.405	0.257	0.756	0.484
Cu_*fruit*_ = 79.912 + 0.149 × Cu_*soil*_ − 7.717 × pH + 0.839 × OM	0.829	0.670	0.059	1.174	0.293
Fe_*fruit*_ = 6102.976 + 0.020 × Fe_*soil*_ − 709.813 × pH − 34.300 × OM	0.603	0.680	0.459	1.877	0.119
Mn_*fruit*_ = 773.484 + 0.047 × Mn_*soil*_ − 86.666 × pH + 11.810 × OM	0.833	0.858	0.121	1.406	0.254
Ni_*fruit*_ = 54.621 + 0.104 × Ni_*soil*_ − 6.260 × pH + 0.057 × OM	0.900	0.642	0.112	1.046	0.343
Pb_*fruit*_ = 18.752 − 0.093 × Pb_*soil*_ − 2.072 × pH − 0.501 × OM	0.873	0.489	0.343	0.076	0.942
Zn_*fruit*_ = 302.152 + 0.369 × Zn_*soil*_ − 32.010 × pH − 2.170 × OM	0.750	0.656	0.079	0.816	0.452

ME: model efficiency, MNAE: mean normalized average error.

## Discussion

### Characteristics of soil-sewage sludge

Soil characteristics such as soil pH, clay, organic matter content and type, and moisture content determine the availability of trace metals to plants by controlling trace metal speciation, the temporary binding of trace metals by particle surfaces (adsorption-desorption processes), precipitation reactions and the availability of trace metals in soil solution^9^. The soil-sewage sludge combination supporting faba bean cultivation was alkaline with pH values ranging from 8.39 to 9.04, which reduces the availability of trace metals as reported by Eid *et al*.^5^ and Galal *et al*.^36^. High soil pH is known to enhance the adsorption of many trace metals, thus decreasing its solubility in soil solutions^24,45^. Soil trace metals such as Fe, Mn, Zn and Cr presented the highest levels in the studied soils, while Co, Pb and Cd were the lowest. According to Jones and Jacobsen^46^, Fe and Mn are the most plentiful trace metals in the lithosphere, and they commonly occur as Fe-Mn oxides and hydroxides, which play a significant role in the precipitation or solubility of some trace metals in soils.

### Trace metal contents in plant tissues

The accumulation of trace metals in different plant organs depends on their form, water transport, and plant species^47^. The current investigation revealed that the different tissues of faba bean accumulated most of the investigated trace metals, except Al, Co and Pb, in their roots rather than in the other tissues; this goes in line with many studies reporting that trace metals are largely retained in roots^1,4,5,7–9^. The high accumulation of trace metals in roots may be ascribed to complexation of trace metals with the sulfhydryl groups, creating in less trace metal translocation to shoot system^48^. In addition, there are many reports on the synthesis of phytochelatins, which can sequester trace metals; therefore, greater accumulation occurred in the roots^15^. Furthermore, higher trace metal concentrations in the roots than in the shoot could be also because roots are the first target organ to come into contact with these trace metals^47^.

Some trace metals are essential to plants at low concentrations (e.g. Zn, Mn and Cu), but when present in high concentrations, it represent a toxic risk to living organisms^21^. In the present study, the highest mean concentration of trace metals in the roots was recorded for Fe, followed by Mn, Cr, Zn, Ni and Cu (15492.1, 555.6, 133.6, 102.8, 57.6 and 27.7 mg kg^−1^, respectively). Meanwhile, the highest concentrations of Al, Co and Pb (17811.3, 46.6 and 35.1 mg kg^−1^) were found in the plant leaves. Generally, most trace metal concentrations in various tissues of broad bean plants were inside the permissible limits and did not overcome the maximum levels of phytotoxic that were reported by Kabata-Pendias^49^. In the current study, the lowest concentrations of Cu, Mn and Zn were recorded in faba bean stems, which play the role of a transfer organ^50^, thus minimum concentrations of these trace metals were found in their cells. On the other hand, the current investigation indicated that leaves had the highest concentration for Al, Co and Pb. The translocation of these trace metals into leaves could be a tolerance mechanism of faba bean plants to sequester Al, Co and Pb in tissues that undergoing shedding. In addition, most investigated trace metals contributed to a lower concentration in the edible fruits; this is supported by Eid *et al*.^4,5^, who recorded relatively low concentrations of trace metals in the edible vegetable parts.

### Correlation coefficient

The present work indicated that the trace metal concentrations investigated in the different tissues of faba bean were positively correlated with the soil organic matter content but negatively correlated with the soil pH. The soil pH is essential factor that controls the availability of trace metals to plants^51^. In addition to soil pH, the soil organic matter content is one of the most vital soil factors affecting trace metal availability^52^. The organic matter content is considered a primary sorbent of trace metals due to its high sorption affinity^53^. It has a substantial role in determining the availability and mobility of trace metals in soils because it could reduce the bioavailability of trace metals in soils by adsorption or creating stable complexes with humic substances^54^. In addition, the organic matter content contributes to providing organic chemicals to the soil solution that may work as chelates and raise trace metal availability to plants^22^. This could partially explain the positive correlation of extractable metals with organic matter contents observed in the present and previous studies^55^. Furthermore, there are significant positive correlations between the concentration of most trace metals in the soil and faba bean plants, supporting the utilization of this plant as a bioindicator for trace metals in contaminated soils^56^. Moreover, it was found that some of the correlation coefficients between soil trace metal concentrations and the corresponding plant trace metals (soil Co with fruit Co; soil Cr with fruit Cr; and soil Fe with stem Fe) were insignificantly positive. This finding implies that plants do not uniformly absorb all of the trace metals in the soil, and even absorption is not a concentration-reliant occurrence for all of the trace metals^9^.

### Transfer factor

As indicated in the study of Eid *et al*.^57^, factors affecting trace metals accumulation by plants could be biological (e.g., species, plant age, physiology, phenology and generation time) or non-biological (e.g., temperature, season, salinity, pH). Basically, considering the trace metal distribution in faba bean tissues, the majority of both essential and non-essential trace metal concentrations were respectably higher in roots than in shoots, which suggested a restricted internal transport of trace metals upward from roots to shoot system. This is described by the compartmentalization and translocation procedures within the plants vascular system^58^. Moreover, the high roots trace metals accumulation may be referred to complexation of trace metals with the sulfhydryl groups, generating in less trace metal translocation to shoots^48^. In the current study, faba bean had TF less than one for all trace metals except Al, Co and Pb. This result was coincided with study of Latare *et al*.^59^ which reported that Pb tends to accumulate poorly in plants, and plant uptake, if it occurs, most of the absorbed Pb is located within the root system of the plant. The differences in values of TF could be correlated to the trace metal interactions which can be originated by conflicting and synergetic processes^60^. Thus, such interactions could affect the efficiency of trace metals uptake and alter their distribution^61^. Plant physiological factors, differences in the solubility and availability of each trace metal ion, and the plant regulation mechanisms to control shoot system concentrations could be other reasons for the different translocation of trace metals^58,62^. Furthermore, the current research showed that Cr, Fe and Pb had the least translocation potential from the roots to the edible fruits of faba bean. Similar results reported that Cr and Pb were the least transferable of all the studied trace metals^53^. However, the overall pattern of bottom to top concentration decrease detected here is consistent with earlier observations of crop fruits being less contaminated than stems and root concentrations being markedly higher than leaf concentrations^63,64^. According to Fu *et al*.^65^, faba bean is sensitive to trace metals and has a low transfer ability for trace metals. In the present study, the concentration of Fe in the soil was high, in contrast to the respective plant concentrations, and this was reflected in a low translocation factor for this metal. A similar results were reported by Eid *et al*.^4,5,7^ for Fe in spinach, cucumber and wheat.

### Health risks

The potential health risk of trace metals from consumption of faba bean was assessed based on the health quotient (HQ). The HQ of Cd and Pb was >1, indicating the potential for an adverse effect to human health that requires more strict control^66^. As the HQ of most of the trace metals investigated, except Al and Mn, were within the safety threshold of 1 for food security, a low health risk can be suggested for trace metal exposure by ingesting faba bean amended with sewage sludge. The HQ of the trace metals in faba bean, as a fruit crop, was lower than in rootstalk and leafy vegetables^67^. Moreover, the total potential risk posed by a mixture of trace metals (Hazard index: HI) was assessed by adding the HQ of each trace metal^42,43^. Based on the probability distribution of HI for total trace metals caused by the consumption of faba bean, the mean HI value was >1. The results implied that excessive consumption of faba bean amended with sewage sludge might pose a potential health risk to consumers. These results are more or less consistent with Hu *et al*.^67^, who reported that fruit vegetables with HI < 1 pose lower health risks than leafy vegetables.

### Regression models

The translocation factor is a rough estimate for the range of trace metal uptake, but it reflects less details about site conditions^22^. On the other hand, the regression models are useful tools for estimating the trace metal concentrations in plants based on soil properties^68^, and they are highly particular to different plants, different trace metals and specific environmental properties^53^. Soil properties, such as pH, clay and organic matter content, and other trace metal concentrations, are well known to have a great influence on the trace metal concentration in plants^28^. Nakamura *et al*.^29^ suggested soil pH as an appropriate variable in the regression models as the soil pH is interrelated to the solubility and ionic state of the trace metal in soil where trace metals are easily leached from acidic soils. As demonstrated in our results, the combination of soil pH and organic matter content may significantly enhance the estimation of trace metal uptake by plants^69^; hence, it is appropriate to be included in prediction models^70^. Up to our knowledge, there are no prediction models for the uptake of trace metals by faba bean in the literature, so we could not compare our results with previous related studies. In the current study, the application of regression models to predict the values of plant trace metal concentrations proved to be an appropriate option because in all cases significant correlations were reported among the trace metal content in soil and soil properties (pH and organic matter content) with the trace metal concentration in plants. These results were contrasted with the studies of Wang *et al*.^71^ and Lopes *et al*.^70^, in which no significant or poor correlations were obtained between plant trace metals and soil trace metals or pH and organic matter content. All of the developed models were significant, and acceptable coefficient of determination (*R*^2^) values were obtained in all cases, ranging between 0.506 for fruit Cr and 0.909 for root Cu. On the other hand, the possible reasons for the relatively low *R*^2^ explained by the models for some trace metals (e.g. Cr and Fe in root) possibly due to the relative low number of replica compared with the previous studies. Additionally, our developed models had a low mean normalized average error (MNAE) ranging between 0.029 and 0.459. According to Hough *et al*.^72^, MNAE below or up to 0.50 indicates that the model concentration is in a similar range as the measured values. Additionally, they reported that the correlations between the measured and predicted values, with a low MNAE, indicated a good fit of the model.

## Conclusion

The present study was conducted to develop a novel regression models for predicting the concentrations of trace metals in faba bean plants from their concentration in the soil by using the organic matter content and soil pH as co-factors. The trace metal concentrations in the different tissues of faba bean showed that most of the investigated trace metals were accumulated in the plant roots rather than in the other tissues. The fruits of faba bean accumulated the lowest concentration of most trace metals except Cu, Mn and Zn, which were found in the plant stems. The trace metal concentrations of the faba bean plants had a significant positive correlation with the organic matter content and a significant negative correlation with the soil pH. Transfer of trace metals from the soil to faba bean roots indicated that Al, Cu, Pb and Zn had a transfer factor that exceeded one, whereas the TF of the investigated trace metals from the roots to the fruits did not exceed one. The daily intake rate of the investigated trace metals did not exceed one in both adults and children. On the other side, the hazard quotient of trace metals from consuming faba bean fruits had values <1 for most investigated trace metals except Al and Mn in adults and children. Moreover, the hazard index from consuming faba bean was found to be 6.01 and 6.91 for adults and children, respectively. The results implied that excessive consumption of faba bean amended with sewage sludge might pose a potential health risk to consumers. Correlations between the measured and predicted trace metal values, with high coefficient of determination and low mean averaged errors reflected the goodness of fit of the model. As well, presence of non-significant difference (*P* > 0.05) between the measured and predicted concentrations of the trace metal means good performance of the developed model. Thus, these models will be useful for prediction of trace metals uptake by faba bean so possible human risks can be identified.

## Supplementary information

Supplementary material 1
