# Supplementary material for: Regression models for monitoring trace metal accumulations by Faba sativa Bernh. plants grown in soils amended with different rates of sewage sludge
Source: Sci Rep. 2019 Apr 1;9:5443. doi: 10.1038/s41598-019-41807-9 (PMC6443791; doi:10.1038/s41598-019-41807-9)
Supplement: Supplementary file 1 — Supplementary material 1 [file 41598_2019_41807_MOESM1_ESM.pdf]

Regression models for monitoring trace metal accumulations by *Faba sativa* Bernh. plants grown in soils amended with different rates of sewage sludge

Ebrahem M. Eid[1,2,*], Sulaiman A. Alrumman[1], Tarek M. Galal[3], Ahmed F. El-Bebany[4]

[1]*Biology Department, College of Science, King Khalid University, Abha 61321, P.O. Box 9004, Saudi Arabia*

[2]*Permanent address: Botany Department, Faculty of Science, Kafr El-Sheikh University, Kafr El-Sheikh 33516, Egypt*

[3]*Botany and Microbiology Department, Faculty of Science, Helwan University, Cairo, Egypt*

[4]*Plant Pathology Department, Faculty of Agriculture, Alexandria University, El-Shatby 21545, Alexandria, Egypt*

*Corresponding author. Tel.: 00966 55 2717026; Fax: 00966 17 241 8205.

E-mail address: ebrahem.eid@sci.kfs.edu.eg, eeid@kku.edu.sa, ebrahem.eid@gmail.com

ORCID: http://orcid.org/0000-0003-2452-4469

Supplementary material 1. Selected chemical properties of sewage sludge and cultivated fields soil used in the pot experiment (means ± standard error, $n = 6$) (After Eid et al.[6]).

**Properties**	**Sewage sludge**	**Cultivated fields soil**
pH	6.38 ± 0.01	8.91 ± 0.01
Organic matter (%)	65.1 ± 0.1	1.9 ± 0.1
Al (mg g^{-1})	20.1 ± 0.3	6.3 ± 0.2
Co (mg kg^{-1})	25.9 ± 0.8	45.5 ± 0.8
Cr (mg kg^{-1})	168.1 ± 2.8	244.1 ± 0.2
Cu (mg kg^{-1})	162.6 ± 1.5	23.0 ± 0.4
Fe (mg g^{-1})	24.4 ± 0.3	24.1 ± 0.1
Mn (mg kg^{-1})	560.7 ± 6.2	740.4 ± 4.4
Ni (mg kg^{-1})	138.7 ± 2.6	63.7 ± 0.4
Pb (mg kg^{-1})	671.1 ± 4.4	25.5 ± 1.4
Zn (mg kg^{-1})	667.6 ± 8.5	70.6 ± 0.7
